# Elicitor-Mediated Enhancement of Secondary-Metabolite Biosynthesis in *Juniperus* (Cupressaceae): Current Advances and Future Perspectives

**DOI:** 10.3390/plants15172593

**Published:** 2026-08-25

**Authors:** Aisulu Orken, Dilnur Tussipkan, Shuga A. Manabayeva

**Affiliations:** 1 Plant Genetic Engineering Laboratory, National Center for Biotechnology, 13/5, Qorghalzhyn Hwy., Astana 010000, Kazakhstan; orkena23@gmail.com (A.O.); tussipkan@biocenter.kz (D.T.); 2 Faculty of Natural Sciences, L.N. Gumilyov Eurasian National University, Astana 010000, Kazakhstan

**Keywords:** *Juniperus* (Cupressaceae), secondary metabolites, tissue culture, elicitors, metabolite biosynthesis

## Abstract

*Juniperus* species are a rich source of pharmacologically important secondary metabolites, including terpenoids, lignans, flavonoids, and phenolic compounds. However, the commercial utilization of these metabolites is limited by the plant’s slow growth, poor natural regeneration, and environmental variability affecting metabolite accumulation. Integrating plant tissue culture with elicitation strategies is a promising approach for sustainably producing these valuable compounds. This review summarizes the diversity, biological activities, and biotechnological significance of secondary metabolites in *Juniperus*, focusing on tissue culture systems and enhancement of metabolite biosynthesis through elicitors. It critically evaluates the current knowledge of the effects of signaling compounds such as methyl jasmonate (MeJA), jasmonic acid (JA), and salicylic acid (SA), as well as other abiotic and biotic elicitors including silver nanoparticles (AgNPs), chitosan, and chito-oligosaccharides. Particular attention is given to their effects on podophyllotoxin, phenolic, flavonoid, and other bioactive metabolite accumulation. Elicitation responses appear to depend strongly on species, culture system, elicitor type, and treatment conditions. This review also highlights major knowledge gaps, particularly the limited understanding of the regulatory mechanisms controlling secondary-metabolite biosynthesis in *Juniperus* species. Overall, the available evidence suggests that integrating optimized elicitation strategies with transcriptomics, metabolomics, and metabolic engineering could improve our understanding of secondary metabolite regulation and facilitate the development of sustainable *Juniperus* tissue culture platforms for producing high-value natural products.

## 1. Introduction

The *Juniperus* L. genus (Cupressaceae) has long been used in traditional medicine as a diuretic, antiseptic, and anti-inflammatory remedy [1]. Recent phytochemical and pharmacological studies have revealed remarkable diversity in the secondary metabolites of *Juniperus* species. These include monoterpenes, sesquiterpenes, diterpenes, lignans, flavonoids, biflavonoids, and phenolic acids. Many of these compounds exhibit antimicrobial, antioxidant, anti-inflammatory, and anticancer activities. [2]. One valuable compound is deoxypodophyllotoxin, an important natural precursor for semisynthetic production of the anticancer drugs etoposide and teniposide [3]. Other pharmacologically important metabolites, such as ferruginol, totarol, sugiol, and β-caryophyllene, have attracted significant interest due to their broad-spectrum activities, including antimicrobial, antioxidant, anti-inflammatory, and anticancer [4].

The commercial exploitation of these metabolites is currently limited by the slow growth, long life cycle, and poor natural regeneration of *Juniperus* species. Substantial variation in metabolite accumulation also results from environmental factors, such as altitude, climate, soil composition, and plant age [5,6,7]. Consequently, the sustainable and consistent production of standardized phytochemical raw materials from natural *Juniperus* populations remains a major challenge [8]. Plant tissue culture is a promising alternative for producing these valuable secondary metabolites sustainably [9,10]. Among the available biotechnological approaches, elicitation has emerged as one of the most effective strategies for enhancing secondary-metabolite biosynthesis [11,12].

Several reviews have addressed the phytochemistry and pharmacological properties of *Juniperus* species [13]. Currently, however, a comprehensive synthesis of the knowledge on enhancing secondary-metabolite production through elicitor signaling in *Juniperus* tissue cultures is lacking [2]. Additionally, recent advances in elicitor signaling, transcriptomics, metabolomics, and metabolic engineering have yet to be discussed in the context of *Juniperus* biotechnology. This review aims to (i) systematically summarize the diversity, biological activities, and biotechnological significance of secondary metabolites identified in *Juniperus* species; (ii) critically evaluate the establishment of *Juniperus* tissue culture systems and the application of elicitor-based strategies to enhance secondary-metabolite production; (iii) assess the effectiveness, advantages, limitations, and current evidence gaps associated with different classes of abiotic and biotic elicitors; (iv) integrate current knowledge on the molecular mechanisms underlying elicitor-induced secondary metabolite-biosynthesis, emphasizing the lack of experimental evidence for *Juniperus*-specific signaling pathways, transcriptional regulation, and regulatory networks; and (v) identify future research priorities, including applying transcriptomics, metabolomics, metabolic engineering, genome editing, and other advanced biotechnological approaches to improve secondary-metabolite production in *Juniperus* species.

## 2. Taxonomy, Distribution, and Morphology of *Juniperus* Genus

The *Juniperus* genus belongs to the Pinales order, the *Cupressaceae* family, and the *Junipereae* tribe [14,15]. Current taxonomic studies recognize approximately 75 species, which are classified into three monophyletic sections: *Sabina*, *Caryocedrus*, and *Juniperus* (*Oxycedrus*) [16]. Most *Juniperus* species are diploid (2n = 2x = 22) with genome sizes ranging from 21.81 to 30.30 pg/2C. Tetraploid species (2n = 4x = 44) have genome sizes ranging from 46.29 to 50.70 pg/2C. The only known hexaploid species is *J. foetidissima* (2n = 6x = 66), with a genome size of 70.58 pg/2C [17].

**Figure 1:**
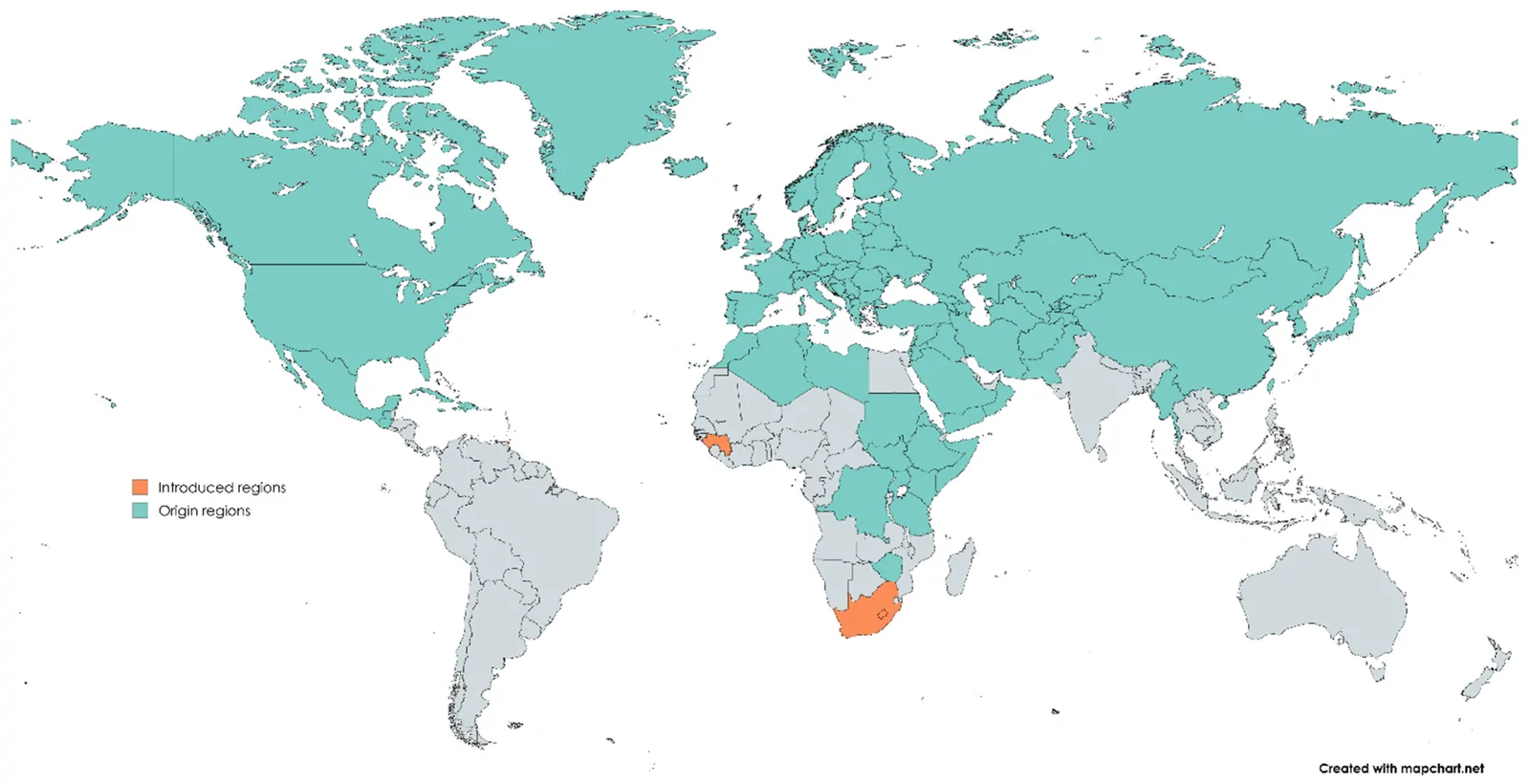
Global distribution of genus *Juniperus*.

*Juniperus* species are distributed almost exclusively throughout the Northern Hemisphere (Figure 1). Most species prefer calcareous soil, though many also grow on granite, sandy, and sandstone substrates. They occupy a wide range of habitats, including deserts, rocky slopes, bogs, and wetlands [18]. Based on phylogenetic, phenotypic, and climatic evidence, the section *Caryocedrus* comprises a single species, *J. drupacea*, which is found in Greece, Turkey, and Syria. The section *Juniperus* contains 14 species, 12 of which are found in the Eastern Hemisphere. *J. jackii*, however, is endemic to North America. The section Sabina comprises approximately 60 species distributed across Asia, Africa, the Mediterranean region, and southwestern North America [19]. *Juniperus* species are ecologically adaptable and occur from sea level to the upper forest limit. Some species reach elevations of 3000–4000 m above sea level [20]. They are highly tolerant of both low temperatures and drought. Experimental freezing studies have demonstrated the complete survival of the buds and needles of *J. communis* at temperatures as low as −60 °C, highlighting exceptional frost tolerance among conifers [21].

In Kazakhstan, the genus *Juniperus* is represented by seven species belonging to the *Juniperus* and Sabina sections [22]. The greatest species diversity is found in the mountainous regions of southern, southeastern, and eastern Kazakhstan. These regions include the Altai, Tarbagatai, Dzungarian Alatau, and Tian Shan mountain ranges. Section *Juniperus* includes *J. communis* and *J. sibirica*, and section *Sabina* includes *J. sabina*, *J. pseudosabina*, *J. semiglobosa*, *J. seravschanica*, and *J. turkestanica*. *J. seravschanica* is listed in the Red Book of Kazakhstan as a rare species with declining populations. *J. semiglobosa* and *J. turkestanica* are considered sub-endemic species of Central Asia [23,24,25].

Morphologically, members of the *Juniperus* genus are evergreen, coniferous shrubs or trees that can grow up to 30 m tall. They are characterized by xeromorphic leaves and fleshy seed cones that are adapted to arid and cold environments [26]. Juvenile plants have needle-like leaves arranged in whorls of three, whereas mature plants have scale-like leaves with resin glands. Female seed cones (galbuli) are globose to ovoid and require one to two years to mature. Each cone consists of one to five fused pairs or trios of cone scales that enclose one to three hard, wingless seeds. The fleshy cones are often resinous and may be bitter or sweet, thereby attracting frugivorous birds that facilitate long-distance seed dispersal by regurgitating or excreting viable seeds [19]. Young shoots are generally slender and terete or occasionally quadrangular and exhibit monopodial branching. This results in highly variable crown architectures ranging from prostrate shrubs to columnar trees. Resting buds are poorly developed or absent. Shoot apices are protected by tightly appressed leaves rather than specialized bud scales, a characteristic feature of the *Cupressacea* family [27]. The secondary xylem consists predominantly of tracheids and axial parenchyma and lacks the typical resin canals that distinguish *Juniperus* from members of the *Pinaceae* family [28]. Growth rings are usually well defined, but they may become extremely narrow under drought conditions or at high elevations. Seedlings exhibit epigeal germination, producing several cotyledons arranged in a whorl, followed by juvenile acicular leaves. The transition from juvenile to adult foliage is gradual and influenced by age and environmental conditions. Furthermore, juvenile needle-like leaves may reappear following pruning, mechanical injury, or environmental stress, reflecting the remarkable morphological plasticity of the genus [29].

## 3. Major Bioactive Compounds of the Genus Juniperus

Many *Juniperus* species are widely used in horticulture, forestry, and traditional medicine due to their ecological adaptability and diverse phytochemical composition [30]. Juniper berries and foliage have long been used as spices and flavoring agents in gin production, as well as in traditional herbal remedies [31]. In horticulture, many *Juniperus* species are cultivated as ornamental evergreen shrubs and trees because they can tolerate drought, nutrient-poor soils, urban pollution, and intensive pruning exceptionally well. This makes them valuable for landscaping, erosion control, and ecological restoration [32]. *Juniperus* extracts exhibit a broad spectrum of bioactivities, including antimicrobial, antioxidant, anti-inflammatory, antidiabetic, and anticancer effects. These effects are largely attributed to the species’ rich diversity of secondary metabolites [8]. For example, *J. procera* produces bioactive diterpenoids such as ferruginol and podophyllotoxin. Both of these compounds have demonstrated pronounced antibacterial and anticancer activities [33]. Phytochemical investigations have revealed that *Juniperus* species synthesize a wide variety of secondary metabolites, including volatile terpenoids (monoterpenes, sesquiterpenes, and diterpenes), as well as phenolic compounds, such as flavonoids (including biflavonoids), phenolic acids, lignans, and coumarins [34]. These metabolites form the basis of many of the genus’ medicinal, pharmaceutical, and commercial applications. For example, essential oils, rich in sabinene, trans-sabinyl acetate, α-pinene, and other terpenoids, have well-documented antiseptic, antimicrobial, and anti-inflammatory properties [34]. Likewise, flavonoids and lignans contribute substantially to the antioxidant and cytotoxic activities of *Juniperus* extracts [33,35].

The secondary metabolites identified in *Juniperus* species can be classified into four major groups based on their chemical structures and biosynthetic origins: monoterpenes, diterpenes, sesquiterpenes, and phenolic compounds [8]. The following sections summarize the major representatives of each group, emphasizing their presence in *Juniperus* species, biological activities, molecular mechanisms of action, and the current state of pharmacological research.

### 3.1. Monoterpenes

*Juniperus* essential oils primarily consist of bicyclic monoterpenes, such as α-pinene, β-pinene, sabinene, and D-limonene (see Table 1). These monoterpenes are the primary volatile components responsible for the genus’s many biological and pharmacological properties [2]. While these monoterpenes exhibit similar biological activities, such as antimicrobial, antioxidant, anti-inflammatory, and anticancer effects, there is growing evidence that they have different molecular mechanisms of action and levels of pharmacological characterization.

Among hydrocarbon monoterpenes, α-pinene is the most extensively studied constituent and has been investigated for its antimicrobial, antioxidant, anti-inflammatory, and anticancer properties [36]. In *Juniperus* oxycedrus, (+)-α-pinene suppresses IL-1β-induced inflammatory responses in human chondrocytes by reducing the expression of inflammatory mediators [37]. Similarly, α-pinene inhibits NF-κB activation and significantly decreases TNF-α and IL-6 production in lipopolysaccharide-stimulated macrophages, demonstrating its ability to modulate key inflammatory signaling pathways [38].

Compared to α-pinene, considerably less mechanistic information is available for β-pinene in *Juniperus* species. Although β-pinene is a major component of several essential oils, including those of *J. communis* and *J. oxycedrus*, most studies have evaluated the biological activities of the oils rather than the purified compound itself. These essential oils exhibit antimicrobial, antioxidant, and antifungal activities. The specific contribution of β-pinene to these effects remains unclear [39,40,41,42]. In contrast, studies using β-pinene isolated from other plant species have demonstrated its selective cytotoxicity toward human oral tongue squamous cell carcinoma cells by inducing caspase-dependent apoptosis. This effect is characterized by membrane blebbing, cell shrinkage, nuclear pyknosis, and attenuation of cell death in the presence of pan-caspase and caspase-3 inhibitors. This indicates the activation of the intrinsic apoptotic machinery [43]. Furthermore, β-pinene enhances the antitumor efficacy of paclitaxel against non-small-cell lung cancer cells, producing a synergistic effect accompanied by chromatin condensation and nuclear fragmentation, which are characteristic hallmarks of apoptosis [44].

D-limonene is another major hydrocarbon monoterpene found in several *Juniperus* species. This compound exhibits antibacterial, antioxidant, anti-inflammatory, and anticancer activities through multiple mechanisms. These mechanisms include modulation of the NF-κB and MAPK signaling pathways and disruption of microbial cell membranes [45]. Taken together, these findings suggest that hydrocarbon monoterpenes are important contributors to the pharmacological properties of *Juniperus* species. However, they also highlight a significant limitation of current research. While detailed mechanistic studies are available for isolated monoterpenes, most research on *Juniperus* species focuses on crude essential oil. This makes it difficult to distinguish the biological activities of individual constituents from potential synergistic interactions among multiple terpenes. Consequently, despite the well-documented bioactivities of α-pinene, β-pinene, and d-limonene, their precise molecular targets and contributions to the biological effects of *Juniperus* extracts remain unclear.

Oxygenated monoterpenes exhibit different biological properties than hydrocarbon monoterpenes [46]. Terpinen-4-ol, a primary component of *J. communis* [47], *J. sabina* [48], and *J. chinensis* [49], has stronger antimicrobial and anti-inflammatory properties than many hydrocarbon monoterpenes. These effects are primarily attributed to its ability to disrupt microbial cell membranes while suppressing NF-κB- and MAPK-mediated inflammatory signaling pathways [50,51].

In contrast, β-myrcene is primarily associated with analgesic and anti-inflammatory activities rather than with direct antimicrobial effects. Its pharmacological activity is mediated by suppressing nitric oxide production and modulating opioid- and adrenergic-dependent signaling pathways [52,53]. This monoterpene is widely distributed in *J. communis* [54] and *J. excelsa* [55].

Several additional monoterpenes, including γ-terpinene, α-terpinene, terpinolene, bornyl acetate, 1,8-cineole, camphor, linalool, and camphene, have been identified in various *Juniperus* species, such as *J. communis* [56], *J. oxycedrus* [56], and *J. phoenicea* [57]. Compared to α-pinene, d-limonene, and terpinen-4-ol, however, these compounds have received considerably less attention. Most available studies describe their antioxidant and antimicrobial activities without providing detailed mechanistic evidence. Therefore, their contribution appears to be complementary rather than dominant. They enhance the overall antioxidant, antimicrobial, and anti-inflammatory properties of *Juniperus* essential oils by scavenging reactive oxygen species, inhibiting lipid peroxidation, suppressing pro-inflammatory signaling, and interfering with microbial biofilm formation [58,59,60,61,62,63].

Overall, monoterpenes are the most abundant and extensively studied volatile constituents of *Juniperus* essential oils. Among them, α-pinene and terpinen-4-ol have the strongest experimental evidence supporting their antimicrobial and anti-inflammatory activities. Despite this, the biological functions of many minor monoterpenes remain poorly characterized. Future research should prioritize comparative studies of isolated compounds, elucidation of their molecular targets, and evaluation of potential synergistic interactions within essential oils to better define their contributions to the pharmacological properties of *Juniperus*.

### 3.2. Diterpenes

*Juniperus* species synthesize a diverse array of bioactive diterpenoids, primarily belonging to the abietane and pimarane classes [64]. However, these compounds have been investigated to markedly different extents. Abietane diterpenoids, particularly ferruginol, totarol, and sugiol, have received considerable attention due to their broad spectrum of biological activities. In contrast, most other diterpenoids have only been examined in a limited number of pharmacological studies.

Totarol, an abietane diterpenoid identified in *J. communis* [2], exhibits broad-spectrum antibacterial activity against both Gram-positive and Gram-negative bacteria by disrupting bacterial membrane integrity and cellular metabolism [65,66]. In addition to its antimicrobial activity, totarol enhances cellular antioxidant defense by activating Akt/GSK-3β/Nrf2 signaling pathway, thereby protecting cells against oxidative stress [67]. While totarol has demonstrated additional pharmacological activities, most studies have focused on its antibacterial properties, comparatively leaving its anticancer potential less explored [68].

Ferruginol is a well-characterized abietane diterpenoid that is widely distributed among *Juniperus* species, including *J. excelsa* [69], *J. procera* [70], and *J. oxycedrus* [71]. In *J. excelsa* berries, ferruginol is one of the major constituents of the hexane extract, accounting for approximately 32.9% of the detected compounds [69]. Extensive pharmacological research has demonstrated that ferruginol exhibits anti-inflammatory, antibacterial, antioxidant, gastroprotective, and anticancer properties [72,73]. Unlike totarol, which has primarily been studied for its antimicrobial effects, ferruginol has been investigated across a much broader range of experimental models. Consequently, it is currently regarded as one of the best-characterized and most pharmacologically versatile diterpenoids identified in the *Juniperus* genus [2].

Sugiol (4α-hydroxy-7-oxo-abieta-8(14),11,13-triene), another abietane diterpenoid isolated from the heartwood of *J. formosana*, has attracted increasing attention due to its antioxidant and anticancer properties [74,75,76]. Experimental studies have demonstrated that sugiol inhibits the proliferation of human ovarian cancer SKOV3 cells by inducing G0/G1 cell cycle arrest and mitochondrial apoptosis. Additionally, sugiol suppresses cell migration by downregulating MMP-2 and MMP-9 expression and inhibits the RAF/MEK/ERK signaling pathway. These findings highlight its ability to target multiple cancer-related signaling networks [77,78]. Although sugiol has been studied less extensively than ferruginol, accumulating evidence suggests that it has considerable therapeutic potential due to its ability to regulate the cell cycle, apoptosis, and tumor cell migration through multiple molecular mechanisms [78,79].

Labdane-type diterpenoids, including pimaric acid, isopimaric acid, and sandracopimaric acid, have been identified in *Juniperus* species. These compounds exhibit antimicrobial and cytotoxic activities [4]. Imbricatolic acid, one of these compounds, has attracted particular attention due to its distinctive pharmacological profile. First isolated from *J. communis* by De Marino et al. [80], imbricatolic acid exhibits remarkable gastroprotective activity and suppresses cancer cell proliferation by inducing cell cycle arrest through the regulation of cyclin-dependent kinase inhibitors [4]. Unlike ferruginol and sugiol, which have been studied across multiple pharmacological models, research on imbricatolic acid has focused on its gastroprotective and antiproliferative effects. While these results suggest significant therapeutic potential, the molecular mechanisms of imbricatolic acid’s action are only partially understood and require further research [81].

Compared to other major abietane diterpenoids, those identified in *Juniperus* species have received considerably less scientific attention, and their biological activities are supported by few experimental studies. For instance, 3α-hinokiol, isolated from *J. przewalskii*, exhibited significant antiproliferative activity against the HeLa cervical carcinoma and HO-8910 human ovarian carcinoma cell lines [82]. Similarly, the abietane diterpenoid 7α-hydroxytotarol, first isolated from *J. brevifolia*, has undergone chemical characterization. Nevertheless, its pharmacological properties remain largely unexplored [83]. These examples illustrate that many *Juniperus* diterpenoids are promising candidates for future pharmacological and mechanistic investigations.

Trans- and cis-communic acids are among labdane-type diterpenoids that are widely distributed throughout the *Juniperus* genus. These resin acids exhibit antimicrobial, antioxidant, and cytotoxic activities, thereby contributing to the biological properties of *Juniperus* extracts and resin fractions [83]. However, their pharmacological activities have not been investigated as thoroughly as those of the major abietane diterpenoids, and the molecular mechanisms underlying their biological effects are not well understood.

Pimarane-type diterpenoids, including sandaracopimarinal, have been identified as minor constituents of *J. excelsa* berry essential oil, representing approximately 0.3% of the total composition [84]. Correlation analyses suggest that sandaracopimarinal may contribute to the essential oil’s antifungal activity. Nevertheless, direct experimental evidence supporting this hypothesis is lacking. Additionally, several abietane resin acids, particularly dehydroabietic acid and abietic acid, have been reported in various *Juniperus* species [85]. These compounds exhibit anti-inflammatory, antibacterial, antioxidant, and gastroprotective activities, highlighting their pharmacological significance [86]. Despite these promising biological properties, most minor diterpenoids remain insufficiently characterized, and their individual contributions to the overall biological activity of *Juniperus* extracts have yet to be clearly established.

Overall, diterpenoids are among the most pharmacologically diverse classes of secondary metabolites identified in *Juniperus* species. Ferruginol, totarol, and sugiol are among the most well-studied and have well-documented antibacterial, antioxidant, anti-inflammatory, and anticancer activities supported by a strong body of experimental evidence. In contrast, most minor abietane, labdane, and pimarane diterpenoids have only been superficially investigated, so their molecular targets, pharmacokinetic properties, and therapeutic potential remain largely unknown. Future studies should focus on elucidating their mechanisms of action, validating their biological activities in vivo, and exploring possible synergistic interactions among individual diterpenoids. This will facilitate their translation into pharmaceutical and biotechnological applications.

### 3.3. Sesquiterpenes

The sesquiterpene fraction of juniper essential oils is characterized by the presence of high concentrations of β-caryophyllene [87,88], α-humulene [89], and several other biologically active constituents [42]. Of these compounds, β-caryophyllene is the most well characterized. Gertsch et al. identified it as the first plant-derived dietary cannabinoid that acts as a selective agonist of the cannabinoid receptor CB_2_ [90]. Activation of CB_2_ receptors by β-caryophyllene exerts pronounced anti-inflammatory and analgesic effects by suppressing pro-inflammatory cytokine production and modulating immune responses [91,92]. In addition to its cannabinoid receptor-mediated activity, β-caryophyllene exhibits broad-spectrum antibacterial, antioxidant, and anticancer properties across diverse experimental models, highlighting its considerable therapeutic potential [93,94]. Compared with other sesquiterpenes identified in *Juniperus*, β-caryophyllene has the strongest mechanistic evidence and is supported by the most comprehensive pharmacological characterization.

α-Humulene commonly co-occurs with β-caryophyllene in *Juniperus* essential oils and has demonstrated anti-inflammatory and antiproliferative activities [95,96]. Despite its structural similarity to β-caryophyllene and frequent co-occurrence, substantially fewer mechanistic studies have been conducted on α-humulene. Consequently, its molecular targets and signaling pathways are less well defined, which limits a comprehensive evaluation of its therapeutic potential.

Another abundant sesquiterpene, germacrene D, exhibits antimicrobial antioxidant, and immunomodulatory activities [97]. Purified germacrene D has been shown to suppress neutrophil activation by inhibiting intracellular Ca^2+^ mobilization and chemotaxis, indicating its anti-inflammatory potential [98]. Nevertheless, existing evidence is predominantly derived from in vitro studies, and confirmation of these biological effects under physiological conditions is still required.

δ-Cadinene has been identified in several *Juniperus* species [99,100] and exhibits potent antibacterial activity, particularly against *Streptococcus pneumonia*. This suggests that inhibiting bacterial growth is one of its primary biological functions [101]. Despite these promising findings, pharmacological investigations of δ-cadinene remain relatively limited, and its broader biological activities, molecular targets, and therapeutic applicability have yet to be characterized comprehensively.

Another representative sesquiterpene, α-cadinol, has been identified in several *Juniperus* species, including *J. communis* [102], *J. wallichiana* [103], and *J. oxycedrus* [104]. α-Cadinol exhibits antimicrobial activity against Gram-positive bacteria and cytotoxic effects toward several human cancer cell lines, highlighting its antimicrobial and antiproliferative potential [105]. However, the available evidence is primarily based on cell-based assays, so additional in vivo studies are needed to determine whether these biological activities can be translated into clinically relevant therapeutic effects.

Cedrol has attracted increasing attention due to its immunomodulatory and anticancer properties. Mechanistic studies have demonstrated that cedrol suppresses neutrophil chemotaxis and induces mitochondrial apoptosis by inhibiting the AKT/ERK/mTOR and NF-κB signaling pathways [106]. While these findings provide stronger mechanistic support than is currently available for many other *Juniperus* sesquiterpenes, information regarding cedrol’s pharmacokinetics, bioavailability, and long-term safety is limited.

Collectively, sesquiterpenoids contribute significantly to the pharmacological properties of *Juniperus* essential oils, including their anti-inflammatory, antioxidant, antimicrobial, and antiproliferative activities [107,108,109]. However, the strength of the available evidence varies substantially among individual compounds. Except for β-caryophyllene, most pharmacological data originate from in vitro studies. Well-designed in vivo investigations, pharmacokinetic analyses, and clinical studies are scarce. Furthermore, most biological studies have evaluated crude essential oils rather than isolated sesquiterpenes. This makes it difficult to distinguish the biological contribution of individual compounds from potential synergistic interactions within these complex phytochemical mixtures. Future research should focus on elucidating the molecular mechanisms of less characterized sesquiterpenes, validating their pharmacological activities in vivo, and clarifying the synergistic interactions that may underlie the therapeutic efficacy of *Juniperus* essential oils.

### 3.4. Phenolic Compounds

*Juniperus* species are particularly rich in phenolic constituents, especially bioflavonoids. Some of the most characteristic metabolites of the genus are amentoflavone, bilobetin, cupressuflavone, and hinokiflavone [4,35]. These compounds contribute substantially to the anti-inflammatory, antiviral, antioxidant, neuroprotective, and anticancer properties of *Juniperus* extracts. The extent of pharmacological characterization of individual bioflavonoids varies considerably.

Amentoflavone is the most extensively studied biflavonoid identified in *Juniperus* species. Numerous studies have demonstrated its anti-inflammatory, antiviral, antioxidant, neuroprotective, and anticancer activities. These activities are mediated by the modulation of multiple signaling pathways involved in oxidative stress, inflammation, apoptosis, and cell proliferation [110,111]. Compared with other biflavonoids, amentoflavone has been studied in a wider range of experimental models. This has resulted in a more comprehensive understanding of its molecular mechanisms of action and therapeutic potential.

Bilobetin and other structurally related biflavonoids have demonstrated promising biological activities, including inhibiting influenza virus replication and inducing apoptosis in cancer cells [112,113,114]. However, there is limited pharmacological evidence supporting most *Juniperus* bioflavonoids. Their biological activities have predominantly been evaluated in vitro, while comparative in vivo studies and pharmacokinetic analyses are scarce. Furthermore, despite their close structural similarity, the structure–activity relationships that govern the pharmacological properties of *Juniperus* biflavonoids have not yet been systematically elucidated.

In addition to flavonoids, *Juniperus* species are an important natural source of aryltetralin lignans, particularly deoxypodophyllotoxin. This compound is one of the most pharmacologically significant secondary metabolites identified within the genus [2,115]. Deoxypodophyllotoxin is structurally related to podophyllotoxin and is a valuable precursor for the semisynthesis of the clinically important anticancer drugs etoposide and teniposide [116,117]. Consequently, this lignan has attracted considerable attention due to its intrinsic biological activities and pharmaceutical significance. Studies have shown that deoxypodophyllotoxin, when isolated from *Juniperus* communis, induces G2/M cell cycle arrest and apoptosis in human breast cancer cells by activating the mitochondrial apoptotic pathway [118]. In addition to its potent anticancer activity, deoxypodophyllotoxin exhibits antibacterial and anti-inflammatory properties, which further emphasize its pharmacological importance [119]. Compared to other phenolic constituents of *Juniperus*, deoxypodophyllotoxin is supported by substantially stronger mechanistic evidence. Currently, despite these promising findings, most of the available data originates from preclinical studies. Further investigations are required to address bioavailability, pharmacokinetics, long-term safety, and therapeutic efficacy in vivo before its full clinical potential can be realized.

Overall, phenolic compounds are a major class of bioactive secondary metabolites in *Juniperus*. They complement the biological activities of terpenoids by exhibiting potent antioxidant, anti-inflammatory, antiviral, and anticancer effects. The strongest experimental evidence supports amentoflavone and deoxypodophyllotoxin, while the pharmacological properties of many other biflavonoids and lignans are not well characterized. Future studies should focus on elucidating their molecular targets, establishing structure-activity relationships, improving pharmacokinetic characterization, and validating therapeutic efficacy in appropriate in vivo models. These efforts will facilitate the development of *Juniperus*-derived phenolic compounds for pharmaceutical and biotechnological applications.

**Table 1:** Major secondary metabolites identified in *Juniperus* species, their biological activities, and principal molecular mechanisms of action.

Representative Compound	Reported Biological Activities	Principal Molecular Mechanisms	References
Monoterpenes			
α-Pinene	Antimicrobial, anti-inflammatory, antioxidant, anticancer	- suppresses NF-κB activation - decreases IL-1β, TNF-α and IL-6 production - inhibits inflammatory signaling	[36,37,38]
β-Pinene	Antimicrobial, antioxidant, antifungal, anticancer	- induces caspase-dependent apoptosis - enhances paclitaxel-induced apoptosis in NSCLC cells	[39,40,41,42,43,44]
D-Limonene	Antibacterial, antioxidant, anti-inflammatory, anticancer	- modulates NF-κB and MAPK signaling - disrupts microbial membranes	[45]
Terpinen-4-ol	Antimicrobial, anti-inflammatory	- disrupts microbial membranes - inhibits NF-κB- and MAPK-mediated inflammatory responses	[47,48,49,50,51]
β-Myrcene	Analgesic, antioxidant, anti-inflammatory	- suppresses nitric oxide production - modulates opioid and adrenergic pathways	[52,53,54,55]
γ-Terpinene, α-Terpinene, Terpinolene	Antioxidant, antimicrobial	- ROS scavenging; inhibition of lipid peroxidation	[56,57,58,59,60,61,62,63]
Diterpenes			
Totarol	Antibacterial, antioxidant	- disrupts bacterial membrane integrity - activates Akt/GSK-3β/Nrf2 pathway	[65,66,67,68]
Ferruginol	Antibacterial, anti-inflammatory, antioxidant, gastroprotective, anticancer	- suppresses inflammatory signaling; - induces apoptosis - scavenges reactive oxygen species	[69,70,71,72,73]
Sugiol	Antioxidant, anticancer	- induces G0/G1 arrest and mitochondrial apoptosis - downregulates MMP-2/MMP-9 - inhibits RAF/MEK/ERK signaling	[74,75,76,77,78,79]
Imbricatolic acid	Gastroprotective, antiproliferative	- induces Go/G1 cell cycle arrest via regulation of cyclin-dependent kinase inhibitors	[4,80,81]
Communic acids	Antimicrobial, antioxidant, cytotoxic	- proposed membrane-associated and antioxidant effects (mechanisms poorly characterized)	[83]
Sandaracopimarinal	Antifungal	- may contribute to antifungal activity of essential oil (correlative evidence)	[84]
Dehydroabietic acid/Abietic acid	Anti-inflammatory, antibacterial, antioxidant, gastroprotective	- anti-inflammatory and antioxidant mechanisms reported, but not fully elucidated in *Juniperus*	[85,86]
Sesquiterpenes			
β-Caryophyllene	Anti-inflammatory, analgesic, antibacterial, antioxidant, anticancer	- selective CB_2_ receptor agonist - suppresses pro-inflammatory cytokines - modulates immune responses	[79,80,81,82,83,84]
α-Humulene	Anti-inflammatory, antiproliferative	- molecular targets remain incompletely characterized	[95,96]
Germacrene D	Antimicrobial, antioxidant, immunomodulatory	- inhibits intracellular Ca^2+^ mobilization and neutrophil chemotaxis	[97,98]
δ-Cadinene	Antibacterial	- inhibits bacterial growth, particularly *Streptococcus pneumoniae*	[99,100,101]
α-Cadinol	Antimicrobial, antiproliferative	- cytotoxicity toward cancer cells - activity against Gram-positive bacteria	[102,103,104,105]
Cedrol	Immunomodulatory, anticancer	- inhibits AKT/ERK/mTOR and NF-κB signaling - induces mitochondrial apoptosis	[106]
Phenolic compounds			
Amentoflavone	Anti-inflammatory, antioxidant, antiviral, neuroprotective, anticancer	- modulates multiple signaling pathways	[110,111]
Bilobetin	Antiviral, antiproliferative	- inhibits influenza virus replication; induces apoptosis	[112,113,114]
Deoxypodophyllotoxin	Anticancer, antibacterial, anti-inflammatory	- induces G2/M arrest - activates mitochondrial apoptosis	[115,116,117,118,119]

## 4. Elicitation Strategies for Enhancing Secondary Metabolite Production in Juniperus In Vitro Culture

### 4.1. Rationale for Elicitation

Elicitation has emerged as one of the most effective biotechnological strategies for increasing the production of valuable secondary metabolites in plant in vitro cultures [11,120]. Elicitors mimic biotic or abiotic stress conditions, activating complex defense signaling networks that redirect cellular metabolism toward the biosynthesis and accumulation of specialized metabolites [121]. This approach is particularly relevant for *Juniperus* species, which are characterized by slow growth, prolonged developmental cycles, and limited capacity for natural regeneration. Furthermore, the qualitative and quantitative composition of secondary metabolites in wild *Juniperus* populations is strongly influenced by environmental conditions, geographical origin, seasonal variation, and plant age [122]. This makes producing standardized phytochemical raw materials challenging. Consequently, applying elicitors to plant tissue culture systems is a sustainable and controllable alternative to harvesting natural populations. This method enables the targeted manipulation of metabolic pathways and enhances the production of valuable terpenoids, phenolic compounds, lignans, and other bioactive metabolites for pharmaceutical, nutraceutical, and industrial applications [123,124].

Elicitors are generally classified into two major groups: abiotic and biotic elicitors [125]. Abiotic elicitors include physical factors such as ultraviolet radiation, temperature stress, drought, salinity, and osmotic stress, as well as chemical agents including heavy metals, nanoparticles, other inorganic compounds, and phytohormone-related signaling molecules. In contrast, biotic elicitors originate from living organisms and include polysaccharides, proteins, glycoproteins, and other molecules derived from microbes or plants that can activate plant defense responses and stimulate secondary metabolism.

### 4.2. In Vitro Culture Platforms for Elicitation

Successful elicitation requires more than an appropriate choice of elicitor. It also necessitates efficient in vitro culture systems that can produce sufficient biomass while maintaining high biosynthetic potential. Studies on secondary-metabolite production in *Juniperus* have predominantly relied on callus and suspension cultures, which remain the primary platforms for elicitation and metabolic engineering experiments. Other systems, such as immobilized cell cultures, have received considerably less attention. Organ cultures, including hairy root cultures, temporary immersion systems, and bioreactor-based cultivation, have not yet been reported. This uneven distribution of research suggests that the available evidence of metabolite production in *Juniperus* is largely based on undifferentiated cell cultures. Meanwhile, alternative production platforms with potentially greater biosynthetic capacity remain unexplored.

Early studies successfully established callus cultures in several *Juniperus* species [126]. Javeed et al. (1980) induced soft, friable callus from the juvenile *J. polycarpos* shoots that were cultured on an MS medium supplemented with 0.5 mg/L of 2,4-D (2,4-dichlorophenoxyacetic acid) and 2.0 mg/L of kinetin [127]. Subsequently, Ilahi (1986) [128] obtained cream-colored callus from shoot cuttings using a two-step culture protocol on an MS medium with different concentrations of 2,4-D and kinetin. However, no shoot regeneration was achieved. Gomez and Segura (1996) [129] demonstrated the morphogenic potential of mature *J. oxycedrus* tissue in vitro. Cytokinins promoted adventitious bud differentiation from cultured leaf explants, with the highest bud differentiation observed on a modified Schenk and Hildebrandt (SH) medium supplemented with BA. The authors also induced somatic embryogenesis from leaf explants of mature trees using media containing 6.0 or 10.0 mM 2,4-D or picloram. In mature *J. oxycedrus* leaf cultures, the frequency of embryogenic callus formation ranged from 6–18% under the tested culture conditions.

Subsequent studies have further expanded the range of *Juniperus* species that can be cultured in vitro. Muranaka et al. (1998) established proliferating callus cultures from the young leaves of *J. chinensis* using an SH medium supplemented with naphthaleneacetic acid (NAA) and kinetin [130]. Callus formation was also induced in *J. excelsa* shoot explants on a modified MS medium containing reduced nitrate salts, 6-benzylaminopurine (BAP), 2,4-D, and glutamine [131]. Baravardi et al. (2014) achieved a 73% callus induction frequency from *J. excelsa* lateral buds cultured on an MS medium supplemented with 3.0 mg/L 2,4-D and 0.2 mg/L kinetin [132]. Similarly, Koçer (2005) reported successful callus induction in *J. communis*, using MS medium supplemented with 0.1 mg/L BAP and 0.5-4.0 mg/L IBA, followed by adventitious bud formation, with 0.1 mg/L BAP and 0.5-4.0 mg/L 2,4-D producing the highest frequency (37.5%) [133]. Embryogenic cultures were subsequently established in *J. communis from* zygotic embryos, whereas *J. procera* produced non-embryogenic cultures, which were further characterized and evaluated for cryopreservation under ABA-containing media [134]. More recently, Salih et al. (2021) [33] reported prolific callus production from *J. procera* seedling explants cultured on Woody Plant Medium (WPM) supplemented with low concentrations of 2,4-D and BAP. Under optimal conditions, they yielded approximately 4.6 g of callus.

Collectively, these studies demonstrate that reliable callus induction protocols have been established for numerous *Juniperus* species using various combinations of auxins and cytokinins. However, significant variation in explant source, basal medium composition, and plant growth regulator concentrations make direct comparisons among published protocols difficult and prevents the identification of universally applicable culture conditions. Furthermore, most studies have primarily focused on biomass production rather than the biosynthesis of target secondary metabolites. Therefore, successful callus proliferation cannot be considered an indicator of enhanced metabolite production, as rapid biomass accumulation often occurs without an increase in secondary metabolism. Future optimization strategies should integrate culture establishment with metabolomic analysis and elicitor responsiveness to identify culture conditions that maximize biomass and phytochemical productivity.

In addition to callus and suspension cultures, organized shoot culture systems have also been successfully established in *Juniperus*. Negussie (1997) [135] demonstrated the efficient induction of adventitious shoots in *J. excelsa* from excised cotyledon segments and embryo explants. The highest shoot proliferation wasobtained on Eriksson medium supplemented with 0.5 mg/L BAP and 0.02 mg/L NAA, where up to 92 adventitious shoots were formed per cotyledon segment. More recently, Fathollahi Qarachoboogh et al. (2025) [136] systematically evaluated several basal media for the direct regeneration and shoot proliferation of *J. excelsa*. Among the tested media, DKW supplemented with 1 mg/L BAP and 0.1 mg/L indole-3-acetic acid (IAA) produced the greatest average number of shoots (8.05 shoots per explant), while hormone-free DKW produced the longest shoots (9.6 mm). The study also demonstrated the successful rooting of regenerated shoots. The highest rooting percentage (33.33%) was obtained on Olive Medium following induction with 0.5 mg/L indole-3-butyric acid IBA. Salih et al. (2021) [33] showed that in vitro shoot cultures of *J. procera* can serve as a source of bioactive secondary metabolites. Shoot multiplication was established using WPM supplemented with 0.5 μM IAA and 0.5 μM BAP, producing approximately 14 shoots per explant. GC–MS analysis of the in vitro shoot and callus extracts revealed more than 20 secondary metabolite-related constituents, including ferruginol. Regenerated shoots could subsequently be elongated and subjected to rooting treatments, demonstrating the feasibility of establishing organized in vitro shoot cultures in *Juniperus*. These differentiated shoot cultures could provide an additional platform for studying and enhancing specialized metabolite production, including through elicitor treatments. However, their application to elicitation and secondary-metabolite production is far less explored than that of callus and suspension cultures.

Despite the development of *Juniperus* tissue culture systems, few studies have investigated enhancing secondary-metabolite production through elicitation. Thus, applying elicitation strategies is a promising way to improve the productivity and commercial potential of Juniperus in vitro cultures.

### 4.3. Abiotic Elicitors

#### 4.3.1. Phytohormone-Related Elicitors

Of the various elicitors investigated in *Juniperus* tissue cultures, phytohormone-related signaling compounds have received considerable attention due to their critical roles in regulating plant defense responses and secondary metabolism. The most extensively studied compounds in this group are jasmonates and salicylic acid (SA), These compounds have been shown to increase the production of pharmacologically important secondary metabolites, such as podophyllotoxin and phenolic compounds.

Methyl jasmonate (MeJA) is one of the most widely used elicitors for stimulating the production of secondary metabolites in plant tissue cultures. Premjet et al. investigated the effects of MeJA, chitopentaose, and their combination on podophyllotoxin production in *J. chinensis* cell suspension cultures. The cultures were maintained at 25 °C under dark conditions with shaking at 120 rpm. The elicitors were added on day 7 of cultivation, immediately before the onset of the exponential growth phase. They defined days 0–7 as the lag phase and days 8–21 as the logarithmic growth phase. Thus, elicitation was initiated at an early defined stage of the growth cycle of the culture. MeJA was applied at a concentration of 10 mg/L, and chitopentaose was used at a concentration of 5 mg/L. MeJA alone increased podophyllotoxin production approximately 1.6-fold compared to the untreated control. However, the combination of MeJA and chitopentaose produced a substantially stronger response, increasing podophyllotoxin production by approximately 16-fold relative to the control (Table 2) [120,137].

Jasmonic acid (JA) has biological effects similar to those of MeJA and can effectively regulate secondary metabolism. Kašparová et al. (2018) [138] evaluated the effects of JA on one-year-old *J. virginiana* var. ‘Glauca’ suspension cultures. The cultures were maintained in a SH liquid medium supplemented with NAA, kinetin, and ascorbic acid at 25 °C under a 16 h light/8 h dark photoperiod with a 21-day subculture interval. Elicitation was initiated on day 14 of the culture cycle, corresponding to the mid-exponential growth phase. JA was applied at concentrations of 0.005, 0.05, 0.5, or 5 mmol/L for 6, 24, 48, or 168 h. Three independent experiments were performed for each treatment. The highest podophyllotoxin content (0.67 mg/g DW) was obtained with 5 mmol/L of JA after 168 h of exposure. This corresponded to a statistically significant increase of approximately 179% relative to the control (Table 2).

Taken together, these studies suggest that jasmonate-based elicitation substantially increases podophyllotoxin accumulation in *Juniperus* species. The stronger response observed following the combined application of MeJA and chitopentaose suggests that the simultaneous activation of jasmonate-mediated and biotic elicitor-responsive pathways enhances metabolite accumulation more effectively than MeJA treatment alone. Nevertheless, the apparent synergistic effect requires further investigation across different *Juniperus* species and culture systems.

Salicylic acid is another signaling compound that has the potential to enhance the production of secondary metabolites in *Juniperus* tissue cultures. In the same study, Kašparová et al. (2018) [138] evaluated SA using one-year-old *J. virginiana* var. ‘Glauca’ suspension cultures under the aforementioned culture conditions. SA was applied on day 14 of cultivation, which corresponded to the mid-exponential growth phase. It was applied at concentrations of 0.01, 0.1, 1, and 10 mmol/L for 6, 24, 48, or 168 h. The highest podophyllotoxin content (0.52 mg/g DW) was obtained with 10 mmol/L SA after 24 h of exposure. This represented a statistically significant increase of approximately 108% relative to the control. In contrast to JA, which produced its maximum response required 168 h of exposure, SA produced its maximum effect after only 24 h. Longer exposure periods resulted in lower podophyllotoxin levels (Table 2). Similarly, Dorri et al. (2020) [139] investigated the effect of SA on secondary metabolism in *J. excelsa* callus cultures. Callus was induced on WPM using various auxin–cytokinin combinations. Complete callus induction was achieved after approximately six weeks. The elicitation experiment was conducted using SA at 50 and 100 μM. Treatment with 100 μM SA produced the highest levels of 31.8 mg gallic acid (GAE) g^−1^ DW, flavonoids 9.3 mg quercetin (QE) g^−1^ DW, and DPPH radical-scavenging activity (35.06%). In contrast, treatment 50 μM SA did not significantly affect total phenolic content compared to the control (Table 2).

Available evidence suggests that jasmonates and salicylic acid can effectively increase podophyllotoxin and other secondary metabolites in *Juniperus* tissue cultures, though their effects depend heavily on the experimental conditions. Of the reviewed studies, the strongest increase in podophyllotoxin production was observed in *J. chinensis* suspension cultures treated with the combination of MeJA and chitopentaose, resulting in a 16-fold increase compared to the control group [120]. However, this finding does not suggest that MeJA is universally superior to other elicitors because the available studies differ substantially in *Juniperus* species, culture systems, culture age, subculture interval, physiological growth phase at the time of elicitation, elicitor concentration, and exposure duration. Even within the same *J. virginiana* suspension culture system, JA and SA showed distinct optimal responses. JA produced its maximum effect at 5 mmol/L after 168 h, whereas SA was most effective at 10 mmol/L after 24 h [138]. These results imply that the elicitation responses are determined by the interaction among the type, concentration, and exposure duration of the elicitor and the physiological state of the culture rather than by the identity of the elicitor alone. Therefore, optimizing hormonal elicitation for podophyllotoxin production requires considering the entire culture cycle, including subculture interval, culture age and growth phase at elicitor application, dose–response relationships, exposure duration, biomass accumulation, and metabolite productivity. While the available studies demonstrate the potential of jasmonates and SA, the limited number *Juniperus* species investigated, and the variability of experimental conditions currently preclude establishing a universal hormonal elicitation regime to maximize podophyllotoxin production.

#### 4.3.2. Chemical Elicitors

Silver nanoparticles (AgNPs) have emerged as promising nano-elicitors for enhancing the production of secondary metabolites in plant tissue cultures. Salih et al. (2022) [140] investigated the effects of biogenic AgNPs on *J. procera* callus cultures using concentrations of 0, 5, 20, and 50 mg/L. The AgNPs were biosynthesized using *Phoenix dactylifera* leaf extract and characterized by several physicochemical methods before their application to the *J. procera* cultures. AgNP treatment significantly affects secondary metabolite accumulation. Total phenolic content increased from 2.5 to 3.6 mg/g DW (44%) at 50 mg/L, while total tannin content increased from 1.9 to 2.3 mg/g DW (21%). Total flavonoid content reached a maximum of 1.0 mg/g DW following treatment with 20 mg/L AgNPs, corresponding to a 43% increase relative to the untreated control (Table 2). HPLC analysis demonstrated that AgNP treatment increased the accumulation of several bioactive compounds compared to untreated control. Gallic acid, ferruginol, hesperidin, rutin, coumarin, tannic acid, and quercetin increased by approximately 71%, 67%, 61%, 44%, 39%, 29%, and 17%, respectively. These compounds were quantified by HPLC using reference standards and calibration curves, and observed increases were statistically evaluated relative to untreated cultures. The strongest responses for coumarin, tannic acid, quercetin, rutin, gallic acid, and hesperidin were observed at 50 mg/L AgNPs. Ferruginol, however, was evaluated separately at 0, 5, 10, and 50 mg/L.

Although AgNPs were found to substantially increase the accumulation of several phenolic compounds and diterpenes, evidence supporting nano-elicitation in *Juniperus* is limited to one study. Consequently, the effectiveness of AgNPs relative to conventional elicitors cannot yet be reliably assessed. Further studies are needed to optimize nanoparticle concentrations, evaluate long-term culture stability, and determine potential phytotoxic effects. Notably, the response to AgNP treatment varied across metabolite classes. The concentration resulting in the highest total flavonoid content (20 mg/L) differed from the concentration producing the highest total phenolic and tannin contents (50 mg/L), indicating a dose-dependent but metabolite-specific response. Additionally, the study evaluated only *Juniperus* species and a callus culture system, which limits direct comparison with elicitation studies conducted in other culture systems, particularly suspension cultures. The reported increases demonstrate the potential of AgNPs to enhance secondary metabolism in *J. procera*. However, the available evidence is insufficient to establish an optimal concentration for maximizing metabolite production across *Juniperus* cultures.

### 4.4. Biotic Elicitors

#### Chitosan and Chito-Oligosaccharides

Chitosan, a natural polysaccharide, is produced by the partial deacetylation of chitin, which is a structural component of fungal cell walls and arthropod exoskeletons [141]. Although chitosan and its derivatives are widely used as elicitors in many medicinal and aromatic plants [142,143], research on *Juniperus* is limited. One of the earliest studies was conducted by Premjet et al. (2002) [137], who examined the effects of chito-oligosaccharides on stem-derived callus cultures of *J. chinensis* (Table 2). The cultures were maintained at 25 °C in the dark and subcultured every 40 days. Elicitor treatments were initiated 25 days after subculture when the cultures reached the exponential growth phase. This provided a defined physiological state for elicitation. Following treatment, the cultures were incubated for 15–30 days before podophyllotoxin determination. Among the tested chito-oligosaccharides, chitopentaose produced the strongest response, increasing podophyllotoxin accumulation approximately 6.4-fold relative to the untreated control, with a maximum concentration of approximately 510 μg/g DW. The authors also observed that the response depended on the concentration of the elicitor and the growth stage of the culture. This indicates that the physiological state of the culture is an important determinant of elicitation efficiency.

Urbanová (2018) [144] provided further evidence of the effectiveness of chitosan in *Juniperus* tissue cultures by evaluating chitosan elicitation in callus and suspension cultures of *J. virginiana* var. ‘Glauca’ and suspension cultures of *J. virginiana* var. ‘Hetzii’. The cultures were maintained on an SH medium that was supplemented with 3.0 mg/L NAA, 0.2 mg/L kinetin, and 15.0 mg/L ascorbic acid. Chitosan was tested at four concentrations (0.001, 0.01, 0.1, and 1 g/100 mL) and four exposure periods (6, 24, 48, and 168 h), which allowed for an evaluation of the effects of elicitor concentration and exposure duration across different culture systems. Podophyllotoxin content was quantified by HPLC. The strongest response was observed in the ‘Glauca’ cultures. In callus cultures, treatment with 0.001 g/100 mL of chitosan for 24 h resulted in a podophyllotoxin content of 0.210%, which is approximately 320% higher than the control. In suspension cultures, the maximum content of 0.140% was obtained after 24 h of treatment with 0.1 g/100 mL of chitosan, corresponding to a 211% increase relative to the control. Thus, the optimal chitosan concentration differed between callus and suspension cultures, whereas the optimal exposure duration was the same. These results suggest that the response to chitosan depends on the culture system, the concentration of the elicitor and cannot be attributed to exposure duration alone.

Overall, chitosan and chito-oligosaccharides are the biotic elicitors most extensively studied in *Juniperus* tissue cultures. Available studies demonstrate their potential to substantially increase podophyllotoxin accumulation, particularly in callus cultures. However, direct comparisons with hormonal elicitors should be interpreted cautiously because the studies differ in terms of species, culture systems, elicitor concentrations, exposure durations, physiological stages, and methods of expressing metabolite content. Currently, the evidence is limited to a few *Juniperus* species and target metabolites, which underscores the necessity of additional research to ascertain the broader applicability of chitosan-based elicitation.

The available evidence collectively indicates that the efficiency of elicitation in *Juniperus* species depends on the type of elicitor and the culture system used. Jasmonates and salicylic acid effectively stimulate secondary metabolite accumulation, and chitosan, chito-oligosaccharides, and silver nanoparticles have demonstrated considerable elicitation potential. Nevertheless, the limited number of comparative studies and variability in experimental designs currently preclude identifying a universally optimal elicitation strategy for *Juniperus* tissue cultures.

**Table 2:** Effects of different elicitors on secondary-metabolite production in *Juniperus* in vitro cultures.

Elicitor	*Juniperus* Species	Culture Type	Elicitor Concentration and Exposure Time	Main Response	Increase Compared with Control	Reference
Abiotic elicitors						
Hormonal elicitors						
MeJA	*J. chinensis*	Suspension cell culture	10 mg/L	Podophyllotoxin accumulation	1.5–1.6-fold combined MeJA + chitopentaose up to 15–16-fold	[120]
JA	*J. virginiana* var. ‘Glauca’	Suspension cell culture	5 mmol/L 168 h	Podophyllotoxin accumulation	0.67 mg/g DW +179% PTOX	[138]
SA	*J. virginiana* var. ‘Glauca’	Suspension cell culture	10 mmol/L 24 h	Podophyllotoxin accumulation	0.52 mg/g DW +108% PTOX	[138]
SA	*J. excelsa*	Callus culture	100 μM	Total phenolics, flavonoids, antioxidant activity	31.8 mg GAE g^−1^ DW 9.3 mg QE g^−1^ DW DPPH: 35.06%	[139]
Chemical elicitors						
AgNPs	*J. procera*	Callus culture	5–50 mg/L	Phenolics, tannins, flavonoids, individual compounds Phenolics +44%, tannins +21% flavonoids +43%, gallic acid +71%, ferruginol +67%, hesperidin +61%, rutin +44%, coumarin +39%, tannic acid +29%, quercetin +17%		[140]
Biotic elicitors						
Chito-oligosaccharides	*J. chinensis*	Stem-derived callus culture	5 mg/L	Podophyllotoxin accumulation	6.4-fold maximum 510 μg/g DW	[120]
Chitosan	*J. virginiana* var. ‘Glauca’	Callus culture	0.001 g/100 mL 24 h	Podophyllotoxin accumulation	0.210% PTOX +320%	[144]
		Suspension cell culture	0.1 g/100 mL 24 h		0.140% PTOX +211%	

## 5. Discussion and Future Prospects

### 5.1. Current Limitations

Despite considerable progress in establishing tissue culture systems for *Juniperus*, the application of elicitation strategies to enhance the production of secondary metabolites remains relatively unexplored. While callus and suspension cultures have been successfully developed for various *Juniperus* species, such as *J. procera*, *J. polycarpos*, *J. excelsa*, *J. communis*, and *J. chinensis* [126], only a few studies have examined the use of elicitors to increase production of valuable secondary metabolites. Furthermore, to our knowledge, hairy root cultures, co-culture systems, and other advanced in vitro production platforms have not yet been reported for *Juniperus* species. Consequently, while the available studies provide a foundation for in vitro culture development, they offer limited information on the targeted manipulation of secondary-metabolite biosynthesis.

In other medicinal plants, elicitors such as AgNO_3_, CdCl_2_, La^3+^, and jasmonate-related treatments have been shown to enhance the production of valuable secondary metabolites, although responses vary depending on the species of plant, the concentration of the elicitor, and the duration of exposure [145,146,147,148]. These findings provide a rationale for the systematic evaluation of comparable elicitation strategies in *Juniperus*, for which such approaches remain insufficiently investigated.

### 5.2. Knowledge Gaps

Despite the growing interest in the phytochemistry and biotechnology of *Juniperus*, there are still several gaps in our fundamental knowledge which limit progress in both basic and applied research. Firstly, the taxonomic coverage of the studied species is still relatively limited. Many studies have focused on European and East Asian species, such as *J. communis*, *J. chinensis*, and *J. rigida*. In contrast, Central Asian species, such as *J. polycarpos*, *J. semiglobosa*, and *J. turkestanica*, have received considerably less attention. *J. polycarpos* is particularly important ecologically because of its role in preventing soil erosion and stabilizing fragile mountain ecosystems in Iran [126]. While in vitro cultures have been established for this species, information regarding its metabolomic profile, responsiveness to elicitors, and the molecular mechanisms that regulate secondary-metabolite biosynthesis is limited.

A second major knowledge gap concerns the genetic and environmental regulation of secondary metabolism in *Juniperus* species. Previous studies have demonstrated that environmental factors, including climate, altitude, and soil composition, can substantially influence the composition of essential oils and the accumulation of phenolic compounds. For example, the essential oil composition of *J. rigida* varies considerably along elevational gradients and is linked to soil nutrient availability. This indicates a significant impact of genotype—environment interactions on secondary metabolic variation [149]. However, the molecular mechanisms underlying these responses are not well understood. In particular, the transcriptomics and metabolomics of elicitor-treated *Juniperus* cultures have not been sufficiently characterized. Key regulatory components potentially involved in stress-induced metabolite biosynthesis, including transcription factors of the MYC2, WRKY, MYB, and NAC families, as well as microRNAs and epigenetic modifications, have not been characterized experimentally in *Juniperus* species. Similarly, direct experimental evidence linking canonical signaling pathways, including MAPK cascades, Ca^2+^-dependent signaling, ROS, jasmonate-, salicylic acid-, and abscisic acid-related signaling, to elicitor-induced secondary-metabolite production in *Juniperus* is limited. Consequently, mechanistic models of secondary metabolite regulation in this genus are largely hypothetical and require experimental validation.

### 5.3. Future Research Priorities and Technological Perspectives

Overcoming the current limitations of *Juniperus* biotechnology requires integrating advanced plant tissue culture techniques with modern multi-omics approaches and metabolic engineering.

The priority should be to systematically evaluate abiotic and biotic elicitors using established callus and suspension cultures, as well as alternative culture platforms, including adventitious root cultures, where feasible. Adventitious root cultures may also represent a promising platform for the elicitation-based production of specialized metabolites in *Juniperus*. Elicitation with compounds such as methyl jasmonate, salicylic acid, chitosan, and yeast extract has been reported to enhance the accumulation of secondary metabolites in adventitious root cultures of medicinal plants [150,151]. However, this approach remains largely unexplored in *Juniperus* and warrants further investigation. Candidate abiotic elicitors may include heavy metal ions and metal-derived compounds, such as AgNO_3_, CdCl_2_, CuSO_4_, and La(NO_3_)_3_, as well as physical treatments such as ultraviolet radiation and osmotic stress. Signaling compounds such as MeJA, SA, and biotic elicitors such as chitosan and yeast extract, are also promising candidates due to their well-documented roles in plant defense responses and secondary metabolism [124]. Based on their successful application to other medicinal plant species [150,152,153,154], these treatments provide a rational starting point for optimizing *Juniperus* elicitation protocols. However, their optimal concentrations, exposure durations, and combinations require species- and culture-specific experimental validation [11].

Following the optimization of the elicitation strategies, a comprehensive metabolomic profile should be obtained using GC-MS and LC-MS/MS to characterize the changes induced in the levels of mono-, sesqui-, and diterpenes, as well as phenolic acids and flavonoids. At the same time, RNA sequencing-based transcriptomic analyses could identify genes that are differentially expressed in the MEP and MVA pathways, phenylpropanoid biosynthesis, hormone metabolism, receptor-mediated signaling, MAPK cascades, and transcriptional regulation. Instead of relying solely on endpoint measurements, integrated metabolomic and transcriptomic analyses should incorporate multiple sampling time points after elicitor treatment, as transcriptional responses and metabolite accumulation can exhibit different temporal dynamics [155,156,157].

The next stage should shift from descriptive profiling to the functional validation of the candidate genes and metabolic pathways that were identified through integrated omics analyses [158]. Candidate genes should be prioritized based on their association with metabolite accumulation, their position within biosynthetic pathways, and the reproducibility of their expression patterns across biological replicates and, when possible, across *Juniperus* species [159]. For terpenoid biosynthesis, genes encoding key enzymes of the MEP and MVA pathways, including DXS, DXR, and HMGR, together with TPS genes, represent important candidate targets [160]. For phenylpropanoid and flavonoid biosynthesis, PAL, 4CL, and CHS may be prioritized when their expression is associated with phenolic and flavonoid accumulation [161]. Likewise, regulatory genes, including members of the MYC2, WRKY, MYB, and NAC families, could be investigated when their expression patterns show consistent associations with elicitor responsiveness and metabolite accumulation [160,162,163,164]. However, in *Juniperus*, these genes should be regarded as candidate targets rather than established regulators because their specific functional roles in elicitor-induced secondary metabolism remain insufficiently validated.

CRISPR/Cas-based approaches could be used to study the roles of confirmed candidate genes and, ultimately, redirect metabolic flux toward valuable compounds [165,166]. A rational strategy would be to validate individual targets through targeted knockout, promoter modification, or overexpression before attempting to modify several pathway nodes simultaneously [167]. For example, modifying rate-limiting biosynthetic or regulatory genes could be evaluated for its potential to increase target metabolite production without substantially reducing biomass accumulation [168,169]. Such experiments would provide stronger evidence for causal relationships between gene activity and specialized metabolite production than transcript–metabolite correlations alone. However, developing efficient transformation, regeneration, and stable genome editing systems remains a major technical challenge for woody *Juniperus* species [167,170].

Another priority is evaluating overall production efficiency rather than metabolite concentration alone. Therefore, future studies should report metabolite productivity relative to biomass and culture volume, as well as biomass yield and culture cycle duration. Promising culture systems should then undergo bioreactor-scale validation to assess reproducibility and scalability [171,172]. Techno-economic analyses are also necessary to determine if *Juniperus* cultures can be a competitive alternative or complement to other production platforms, such as field cultivation, microbial fermentation, or chemical synthesis, when applicable [10,173]. When genetically engineered or genome-edited *Juniperus* cultures are intended for commercial applications, regulatory feasibility should likewise be considered [174]. Regulatory requirements vary by jurisdiction and depend on the type of genetic modification and intended application. Thus, regulatory compatibility must be evaluated alongside biological performance, scalability, and economic feasibility.

The rationale for developing *Juniperus* as a biotechnological production platform is not based on the exclusivity of its individual metabolites because many pharmacologically relevant compounds found in the genus are produced by other plant species as well. Rather, its potential lies in the presence of valuable lignans and diterpenoids, their accumulation in aerial tissues, and the ability to culture them in a controlled in vitro environment. Podophyllotoxin and deoxypodophyllotoxin are particularly relevant targets because podophyllotoxin is an important precursor for semisynthesizing the anticancer agents etoposide and teniposide. *Podophyllum hexandrum* is a particularly rich natural source of podophyllotoxin, with levels reported at approximately 4.3% of dry weight. However, its conventional exploitation is limited by slow growth, intensive harvesting, and limited organized cultivation due to conservation concerns [175]. In comparison, *J. bermudiana* has been reported to accumulate up to 22.6 mg/g DW podophyllotoxin and 4.4 mg/g DW deoxypodophyllotoxin in leaves. These levels are high among those reported in *Juniperus* leaf extracts, supporting the potential of aerial *Juniperus* biomass as an alternative source [115]. Importantly, *Juniperus* has biotechnological potential beyond its natural metabolite content because its cell cultures respond strongly to elicitation. For example, a combined treatment of chitopentaose and methyl jasmonate increased podophyllotoxin production by about 15-fold in *J. chinensis* suspension cultures, which substantially exceeded the response to either elicitor alone [120]. Therefore, *Juniperus* should not be considered a universally superior replacement for *Podophyllum*, but rather as a complementary, potentially scalable source of high-value specialized metabolites. This is particularly true when elicitation enhances production under controlled culture conditions and reduces dependence on harvesting slow-growing or vulnerable natural populations.

A realistic pathway toward the commercial production of *Juniperus* metabolites should move beyond simple elicitor screening and establish an integrated production pipeline. First, callus and suspension cultures that perform well should be selected for specific target metabolites. Combined MeJA-chitopentaose treatment is a promising starting point for podophyllotoxin production in *J. chinensis*. Second, rather than focusing solely on metabolite concentration, the elicitor concentration, exposure duration, culture age, and biomass productivity should be optimized simultaneously. Third, time-resolved metabolomic and transcriptomic analyses should be used to identify responsive biosynthetic pathways and candidate regulatory genes. Then, the most promising targets should be functionally validated. Finally, the most productive cultures should be transferred to bioreactor platforms and evaluated based on reproducibility, volumetric productivity, culture cycle duration, scalability, and techno-economic feasibility. This stepwise strategy could provide a practical route from laboratory-scale elicitation studies to the standardized, large-scale production of valuable *Juniperus* metabolites.

## 6. Conclusions

This review provides a comprehensive overview of the diversity, biological activities, and biotechnological significance of secondary metabolites produced by *Juniperus* species. It also summarizes the current status of tissue culture systems and elicitor-based strategies for enhancing their production. The available evidence suggests that elicitation is a promising approach for increasing the accumulation of pharmacologically valuable compounds, such as deoxypodophyllotoxin, terpenoids, and phenolic metabolites. The strongest response observed among the reviewed studies was in *J. chinensis* suspension cultures treated with a combination of MeJA and chitopentaose, which substantially increased podophyllotoxin production. Chitopentaose alone enhanced podophyllotoxin accumulation in *J. chinensis* callus cultures. Chitosan increased podophyllotoxin accumulation in callus and suspension cultures of *J. virginiana* var. ‘Glauca’. Other effective treatments included JA and SA for enhancing podophyllotoxin accumulation, as well as AgNPs for increasing phenolic compounds, including gallic acid and ferruginol. However, elicitation studies in *Juniperus* are limited, especially for Central Asian species, and the molecular mechanisms underlying elicitor-induced metabolic responses are not well understood.

Future progress in *Juniperus* biotechnology requires integrating optimized elicitation strategies with advanced multi-omics approaches, such as transcriptomics, metabolomics, functional genomics, and metabolic engineering. These integrated studies could help us identify the regulatory networks specific to each species and culture that are involved in secondary-metabolite biosynthesis. They could also provide us with a mechanistic understanding of elicitor responses and facilitate the development of efficient, sustainable *Juniperus* tissue culture platforms for producing high-value natural products with potential pharmaceutical, nutraceutical, and industrial applications.

## Data Availability

All relevant data have been provided as Tables and Figures in the text.
